# Supplementary material for: Association between oral microbiome and seven types of cancers in East Asian population: a two-sample Mendelian randomization analysis
Source: Front Mol Biosci. 2023 Nov 21;10:1327893. doi: 10.3389/fmolb.2023.1327893 (PMC10702768; doi:10.3389/fmolb.2023.1327893)
Supplement: Supplementary file 1 [file DataSheet1.ZIP › Supplementary Materials/MR plots for tongue/tongue═╝/Colorectal cancer/pheno.2926_to_colorectal cancer_leave_one_out.pdf]

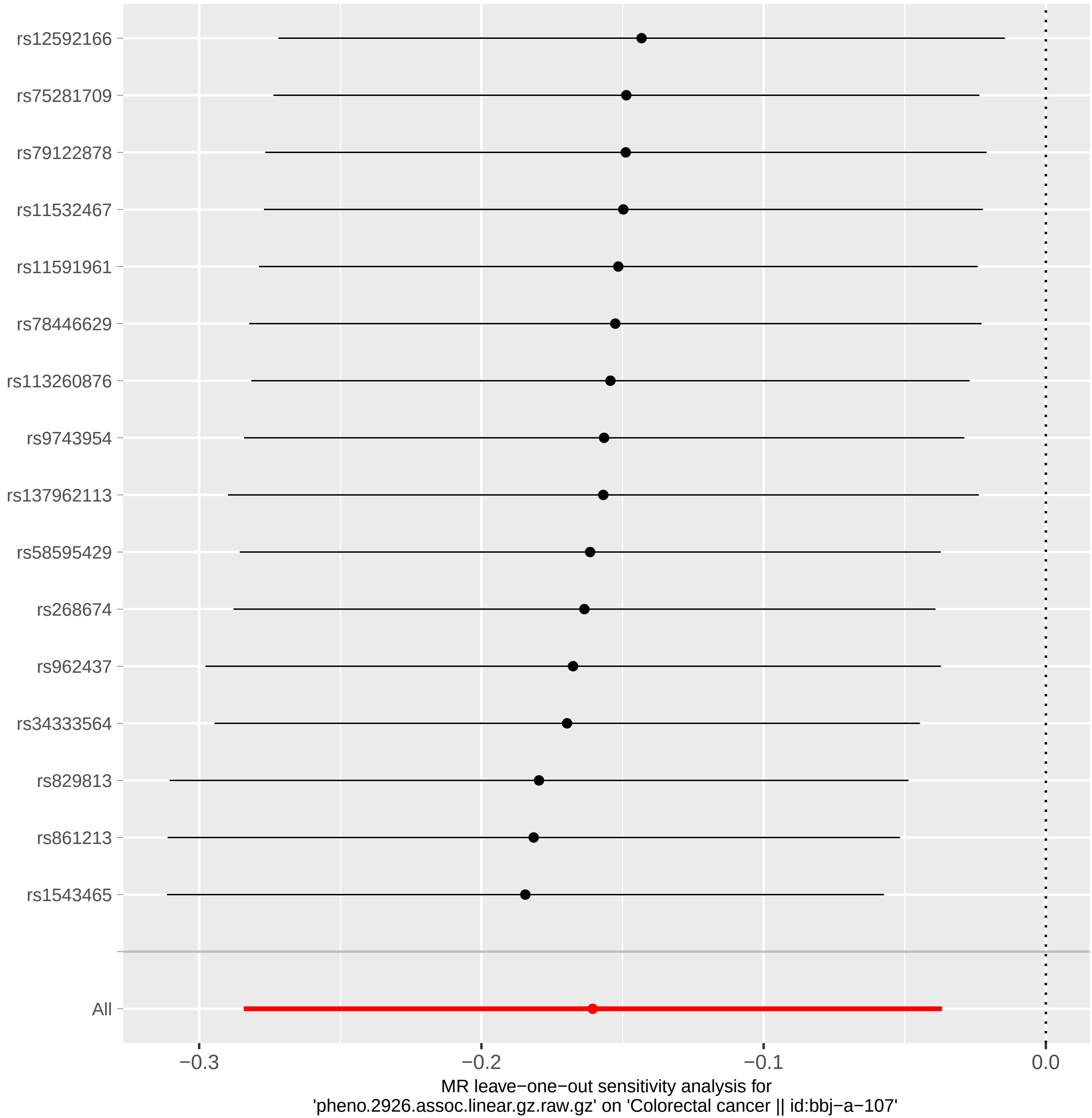

rs12592166
rs75281709
rs79122878
rs11532467
rs11591961
rs78446629
rs113260876
rs9743954
rs137962113
rs58595429
rs268674
rs962437
rs34333564
rs829813
rs861213
rs1543465
All
−0.3
−0.2
−0.1
0.0
MR leave−one−out sensitivity analysis for
'pheno.2926.assoc.linear.gz.raw.gz' on 'Colorectal cancer || id:bbj−a−107'
